# Treating Systemic Lupus Erythematosus (SLE): The Impact of Historical Environmental Context on Healthcare Perceptions and Decision-Making in Charleston, South Carolina

**DOI:** 10.3390/ijerph17072285

**Published:** 2020-03-28

**Authors:** Wendy Rodgers, Edith M. Williams, Brittany L. Smalls, Tyler Singleton, Ashley Tennessee, Diane Kamen, Gary Gilkeson

**Affiliations:** 1 Division of Rheumatology and Immunology, Department of General Internal Medicine, Medical University of South Carolina, 96 Jonathan Lucas Street, Charleston, SC 29425, USA; we.rodgers@gmail.com (W.R.); kamend@musc.edu (D.K.); gilkeson@musc.edu (G.G.); 2 Department of Public Health Sciences, Medical University of South Carolina, 135 Cannon Street, Suite CS303D, Charleston, SC 29425, USA; tylersingleton0921@gmail.com; 3 Department of Family and Community Medicine, University of Kentucky College of Medicine, 2195 Harrodsburg Road, Lexington, KY 40504, USA; brittany.smalls@uky.edu; 4 Center for Health Equity Transformation, University of Kentucky College of Medicine, 760 Press Avenue, Lexington, KY 40508, USA; 5 College of Health Professions, Medical University of South Carolina, 151-A Rutledge Avenue, Charleston, SC 29403, USA; ashley.tennessee@gmail.com

**Keywords:** community-based research, cultural context, lupus

## Abstract

Introduction: Over 400,000 slaves were taken from Africa and brought to Charleston, South Carolina, as part of the transatlantic slave trade during the 18th and 19th centuries. Due to these negative historical events, the healthcare of African Americans in Charleston may be compromised in regard to chronic illnesses and other conditions affecting minorities, such as lupus. Materials and Methods: The current study used an ethnographic approach to obtain the perspectives of lupus patients with the goal of identifying gaps within current research. In addition to patient perspectives, the geographical location of Charleston, South Carolina was considered through inquiries around culture, community, advocacy, and client/patient interaction to establish a narrative for the themes that emerged. Results: The eleven major themes identified were connectedness, knowledge, experience with lupus, compliance, clinical trial participation, career and planning for the future, visits, access to resources, lifestyle, transition from child to adult care, and an overarching theme of self-management. Conclusion: Understanding healthcare perceptions and decision-making among culturally diverse populations, particularly those who have been defined by centuries of substandard care, marginalization, exploitation, and distrust, is critical to the development of culturally tailored interventions designed to improve patient outcomes and reduce health disparities.

## 1. Introduction

The historical inhumane treatment of African Americans in American society, rooted in the history of slavery in America, has molded, framed, and perpetuated the negative thoughts and perceptions of healthcare in America held by members of the African-American community. Beginning in the sixteenth century, Africans were sold from their tribes into slavery by tribal kings for infractions that included theft, murder, or other crimes deemed reprehensible [1]. Many slaves suffered the degradation and humiliation of the middle passage as they were traversed the world in chains, soiled with human waste, often shackled to a corpse, riddled with disease, and if overtaken by diseases, fed to the sharks as they followed the ships across the Atlantic [2]. Of the more than 4 million slaves that arrived in the United States, nearly 10% or 400,000 were brought to South Carolina, specifically, Charleston, South Carolina. Approximately, 40% of slaves who crossed the Atlantic were brought into the Charleston harbor making Charleston, South Carolina the capital of the slave trade [3]. 

Interestingly, most Charlestonian slaves were considered “urban slaves” meaning they were afforded allowances from their owners, such as being allowed to marry, to have children, and were given tasks to complete at their discretion [4]. These allowances were the opposite of the customary conditions to which slaves outside of Charleston were subjected. These allowances were not a manifestation of the good moral character of slave owners but the need for slave owners to capitalize on their ability to effectively farm rice, a lucrative Asian import at the time. As a result, White slave owners began to purchase slaves from the areas where rice was farmed, particularly Sierra Leone. At present, the Gullah people, slaves from Sierra Leone who have inhabited the coastal areas of South Carolina and Georgia known as the “Low Country,” remain in Charleston, South Carolina. 

A study conducted by Jacobs and colleagues [5], which sought to understand the constructs of how African Americans, defined the terms trust and distrust in physicians, found that study participants placed trust in a physician when there was a perception of interpersonal and technical competence of physicians. Contrarily, distrust was defined as a lack of interpersonal and technical competence, perceived quest for profit, and expectations of racism and experimentation during routine provision of healthcare [5]. 

Deeply rooted feelings of distrust have impacted race relations as well as the care-seeking behavior patterns of African Americans including those in Charleston, South Carolina. Dr. Alden Landry, an African-American emergency room physician, recounts the words of a patient who was an older African-American female as he entered her room: “Thank you for being my doctor! I’m so proud of you. I’m glad you’re going to be taking care of me.” Dr. Landry says African-American patients “feel more comfortable with me as their physician” [6]. The reverberating negative psychological impacts of slavery have perpetuated certain beliefs among African Americans. Particularly that quality, reliable healthcare for African Americans is only possible if the doctor providing care is also African American [7]. However, the findings of Malat and van Ryn [8] challenge this belief by indicating that 1 in 5 or 20% of African Americans prefer a same-race provider. Though, this study did not assess knowledge of historical mistreatment nor perceptions of current racial inequities in medical treatment, their preference was the result of personal experiences of discrimination in healthcare.

While provider preference may not be attributed to knowledge of historical mistreatment, research findings reported by Glass and McAtee [9] examine how the human body responds and changes over time after extended exposure to adverse social conditions. The term weathering, coined by Geronimus [10] succinctly encompasses the cumulative changes that occur in bodily systems as a function of repeated exposure to social adversity and embodiment. Researchers Krieger and Davey Smith [11] introduced the term embodiment which “describes the sculpting of internal biological systems that occurs as a result of prolonged exposure to particular environments. It is how features of social and built environments become internalized, or get under the skin” [9].

Beginning in the 1500s and continuing into the present day, African Americans have been exposed to adverse social environments including more than 400 years of slavery, instances of medical exploitation, such as the Tuskegee experiment and Eugenics in North Carolina, as well as recent reports of disproportionately substandard care. Moreover, when compared with their White counterparts Blacks in their 20s, 30s, and 40s are more likely to live with or die from conditions that characteristically occur at older ages [12]. The gravity and extended period of exposure to such traumatic and inequitable contextual circumstances should be considered when examining patient perceptions of care and healthcare seeking behaviors at the onset of disease [12].

In the United States, the highest lupus morbidity and mortality rates are among African-American women [13,14,15]. Systemic lupus erythematosus (SLE) affects approximately 1 in 250 African-American women of childbearing age, and African Americans overall have three to four times greater prevalence of lupus; risk of developing lupus at an earlier age; and lupus-related disease activity, damage, and mortality compared with Caucasians [16,17,18,19,20]. Some have positioned elevated rates of SLE in African-American women in the context of “immune cognition,” and suggest that the disease, for these women, is a physical manifestation of patterns of stress, discrimination, and social disadvantage [21,22,23,24,25,26,27].

Thus, the aim of this study is to examine the impact of the contextual environment in Charleston, South Carolina on the healthcare perceptions and decisions of patients with systemic lupus erythematosus (SLE), providers, and community members.

## 2. Materials and Methods

An ethnographic approach was used to identify themes in interviews conducted at the Medical University of South Carolina (MUSC). Insight is gained through observation and interactions with individuals who share mutual backgrounds [28]. Ethnographic research requires a triangulation of informal and formal interviews, documentation, and observation of individuals to understand culture [28]. This study used an ethnographic approach published by Mendelson [29] involving lupus patients related to assessing disease management. The current study similarly evaluated lupus patients’ perspective to identify gaps within the current research. In addition to patient perspective, the geographical location of Charleston, South Carolina was applied through culture, community, advocacy, and client/patient interaction to establish a narrative for the themes that emerged.

### 2.1. Recruitment and Data Collection

A convenience sample of participants was recruited as part of an immersive summer practicum experience. Participants included patients of the MUSC Rheumatology Clinic who gave permission for their names to be included in MUSC Multidisciplinary Clinical Research Center (MCRC) SLE database, a network-based system of current or former patients, for future clinical research studies. Participants also included providers, community members, and historical tour guides. Guides were included in interviews due to the contextual historical information they could add to conversations. Potential participants were approached in person by the first author and person fulfilling her practicum experience. Once participants provided verbal consent, a conversational interview was conducted at a time and place convenient to the participant, and in a manner that ensured their privacy and confidentiality. All interviews and observations were transcribed and saved as Microsoft^®^ Word files.

### 2.2. Data Analysis

De-identified transcripts were imported into NVIVO 10 for data analysis. Deductive coding identified themes related to healthcare access and perceptions followed by open and NVIVO coding [30]. Codes were compiled and categorized into major themes and subthemes. Thematic coding was conducted independently by E.W. and T.S. Discrepancies in emerging themes were resolved using discussion, prior research, and review of social learning theory. Memoing was also conducted by W.R., E.W., T.S., and A.T. about similarities between prior research, historical context, and interview responses. Triangulation was achieved through thematic coding (triangulation of researchers) and memoing by two researchers [31,32]. The utilization of these triangulation methods allowed for validation of the various perspective and findings from the interview participants as well as researchers in coding [31]. Perspectives gained through analysis and triangulation eliminate the risk of deficiencies from the interviews gathered.

## 3. Results

Unstructured qualitative interviews were conducted with 19 individuals; 9 patients (consisting of 2 Caucasian females, 1 Hispanic female, 5 African-American females and 1 African-American male), 4 rheumatologists (2 Caucasian females, 1 Caucasian male, and 1 African-American female), 4 community members (2 African-American females, 1 identifying as Gullah, 1 African-American male, and 1 Caucasian female), and 5 tour guides: Fort Sumter/Carriage Tour (Caucasian Male), McLeod (African-American Female), Gullah (Gullah Male), and remaining plantations (1 Caucasian Male and 1 Caucasian Female). Verbal informed consent was received prior to proceeding with each interview. All interviews were conducted face to face. Participant interviews (*n* = 9) focused on issues surrounding access to care, perspective of clinical trials, experience living with lupus, and ability to conduct self-care practices. Interviews with providers (*n* = 4), community members (*n* = 4), and tour guides (*n* = 2) expanded on these themes. Ultimately, themes emerged from shared stories, revealing commonalities among patients and community members across their cultures and through experiences attempting to manage their diagnosis.

### Theme 1: Connectedness

In their interviews participants expressed having a support system that believed in the medical treatment as well as their ability to be self-efficient. A feeling of connectedness to their community, as well as the health professional, was essential to staying in positive spirits. Participants believed their lupus would improve and remained confident in navigating the healthcare system with their diagnosis. Furthermore, participants explained that they felt more connected when interacting with health professionals who shared commonalities, such as racial background. Lupus organizations to support patients are scarce, which hinders patients’ ability to feel connected to their community. The city of Charleston is also known as the “Holy City,” due to its large faith-based community. As a result, religion and spirituality were additional elements that tied the individual to their community and its people. Pesut [33] affirmed that elements of spirituality reinforce connectedness in individuals as well as the healthcare profession.

“I wanted them to be here to see this was a good thing. So many of us are afraid, but I believed this was my way to get better … to be a mom again, and I wanted them to believe too.” (when asked about family support in treatment success).

—Interview 1

“I trust Dr. Gilkeson and MUSC doctors”.

—Interview 1

“My faith is everything”.

—Interview 1

“How am I supposed to talk with or calm down those who don’t have quality support and/or the finances to pay their bills?”.

—Interview 7

### Theme 2: Knowledge

Patients stated that explaining their diagnosis and their experiences to others was problematic because they felt a lack of empathy. In addition to knowledge gained about their diagnosis and accompanying medications, patients also learned the importance of incorporating self-care to mentally, emotionally, and physically enhance their well-being. Patients shared that talking about what they have endured to others who can relate to their diagnosis was helpful in passing knowledge. Many patients brought family members to their appointments to receive education and reinforce shared information that may have been forgotten because of their diagnosis.

### Theme 3: Experience (with Lupus)

Participants in interviews stated they had difficulties with their diagnosis because they had not discovered what worked best for themselves on an individual level. Barriers such as mobility, care accessibility, and finances made their experience challenging. In cases where patients had a strong support system, their attitude regarding their lupus translated as more confidence in their ability to self-manage their lupus.

### Theme 4: Compliance

Young adults diagnosed with lupus reported that medication adherence was challenging because of their less-structured lifestyle and forgetfulness. Caregivers played a large role in the ability for younger clients to follow through with their overall healthcare as well as medication regimen. Caregivers active in their role were the driving force behind lupus patients and their motivation to follow through with medical advice given. The environment created at MUSC has also been attributed to medication compliance because of the expectation set through their engagement.

### Theme 5: Clinical Trial Participation

Clinical trials through MUSC were final resorts to help with the management of individual lupus symptoms. Lupus patients’ participation in trials was met with skepticism from their immediate support system (i.e., family members) due to lack of knowledge, but there was an acknowledgement that clinical trials provide hope for a better life with lupus. Throughout the process, individuals with lupus educated those who were unaware of the advantages of clinical trial participation. With hopes of receiving a successful clinical trial, patients are optimistic in regaining their physical mobility, pain management, and daily functioning. Clinical trials served as a resource for many to gain knowledge and additional medical care that they cannot afford.

“When I was in a wheelchair, I couldn’t care for or play with my child … this was the most devastating. I researched clinical trials because I had nothing to lose”.

—Interview 1

“This disease stripped my skin literally and this is how I feel—stripped, but I try to stay positive. If someone can find a cure because I participated, then it is worth it”.

—Interview 9

### Theme 6: Career; Planning for the Future

Lupus has been stated by patients to be the primary deterrent for careers or educational endeavors because of the pervasiveness of the disease. Lupus patients who held a job while diagnosed were most affected by the physical difficulties that accompanied their diagnosis. Financial and family responsibilities are the driving force for patients to maintain a career. Because a lupus diagnosis can be unpredictable, patients are tasked with finding employment that will understand the variability in their day-to-day level of activity. The experiences and interactions throughout a patient’s lupus journey have also been the driving force behind potential career avenues.

“I was in so much pain doing this job”.

—Interview 3

“I kinda gave up because I don’t see what type of work I can do”.

—Interview 4

### Theme 7: Healthcare Visits

Patients used their healthcare visits as a time to connect with their healthcare provider. Individuals diagnosed with lupus often encounter a number of different physicians, which can result in inconsistent interactions. Existing cultural barriers naturally lend to the mistrust that minority patients feel through interactions with medical personnel. Communication and relationship between doctor and patient impacted overall comfort level experienced, as demonstrated by appearance. Doctor visits were also a platform for patients to share their concerns and ask questions in a “safe space.” Visits served as a channel where difficulties with outside providers, such as social workers, and needs could be expressed and possibly met through the resources provided at MUSC.

“I hate to get shots. I don’t what them to put anything into my body … this is what makes people sick”.

—Gullah

### Theme 8: Access to Resources

Due to geographical location, patients reported not having access to reliable medical facilities in their immediate community. Specifically, lupus patients stated that there was a lack of lupus-related treatments and activities. All patients, with the exception of two, who lived outside of downtown Charleston, mentioned having to work on reliable transportation and compromising times with others to access care. Travel times easily exceeded one hour per visit.

Those who lived directly in the community had minimal to no problems accessing their medical needs because of MUSC. In contrast, patients who do not struggle financially to meet their medical needs were still met with difficulty because they felt that they had exhausted treatment options to manage their lupus. For younger patients, scheduling conflicts posed the greatest barrier to their ability to access appointments.

“I couldn’t have found better care. I am supposed to be here”.

—Interview 9

### Theme 9: Lifestyle

Financial and emotional barriers resulting from the treatment of lupus symptoms have posed challenges to the physical well-being of patients. Lupus and its impact on the body are individualized, meaning no two experiences are the same. With lupus, many individuals have had to adapt their lifestyle because of the physical toll that medications have taken on their bodies. Some patients have had to rely on the help of family and friends more than others diagnosed due to the varying level of pain they experience day-to-day that impacts mobility and functioning.

“Years of medications have taken a toll on me”.

—Interview 2

“I don’t have a life with disease; I’ve been in bed most of the time and the pain was unbearable”.

—Interview 2

### Theme 10: Transition from Pediatric to Adult Care

Lupus patients who received their diagnosis in their youth have a harder time making the transition to having full control of their own health decisions. There may have been a lack of knowledge in terms of why certain medications were taken, what they were, and the benefits of taking them. Throughout the course of their diagnosis, caregivers helped to navigate the large and often confusing medical system for them, lessening the role of the patient in the process. Additionally, those who began at a pediatric doctor felt a sense of protection because this trusted healthcare member was a major part of their lupus journey.

### Theme 11: Overarching Theme of Self-Management

The more experienced an individual becomes with the hurdles that accompany their lupus diagnosis; the more information is learned about their individualized self-management process. As patients begin to feel more in control, their level of excitement and engagement increases about their experience. Loss of independence is a factor related to self-management that many patients have deemed frustrating, especially if younger in age.

“I know I can find a way to move forward”.

—Interview 9

## 4. Discussion

This ethnographic study explored healthcare perceptions and decision-making among lupus patients and community members residing in Charleston, South Carolina, a region of the southeastern United States known for its history of racial injustice. The eleven major themes identified were connectedness, knowledge, experience with lupus, compliance, clinical trial participation, career and planning for the future, visits, access to resources, lifestyle, transition from child to adult care, and an overarching theme of self-management. Existing qualitative and quantitative studies have focused on healthcare perceptions and decision-making. However, the current study builds up on this body of knowledge by examining healthcare perceptions and decision-making among Charleston, South Carolina residents to understand the impact of the historical environmental context on perceptions and decision-making in general and related to care of lupus. Understanding healthcare perceptions and decision-making among culturally diverse populations, particularly those positioned in contexts that have been defined by centuries of substandard care, marginalization, exploitation, and distrust, is critical to the development of culturally tailored interventions designed to improve patient outcomes and reduce health disparities.

From observations made on a trip to the Low Country, an area known for its culture, located along the coast of South Carolina, valuable information was gained from interviews with patients living with lupus and the narrative that surrounded their lives. Through information gathered, researchers hope to improve treatment for patients managing lupus, at the Medical University of South Carolina (MUSC) and beyond.

Respondents were self-reflective about how lupus affected their lives, disease self-management, and their social interactions. Similar to other reports, patients described how their level of physical functioning had declined as well as strategies used to regain physical functioning, the longer they had this chronic illness [34,35]. Additionally, they detailed emotional difficulties resulting from clinical manifestations of lupus. Most responses were very personal and recounted individual experiences and struggles that were not directly related to the historical environmental context in Charleston, South Carolina.

Conversely, while many studies have linked historical experiences of racism and exploitation to distrust of the medical care system [36,37], current respondents expressed a strong sense of connectedness with their providers and comfort with the information received from their doctors and their ability, as patients, to voice any concerns they may have. While these findings contradict literature detailing persistently poor patient–provider communication among African Americans [38,39,40,41], they are consistent with previous reports of the unique interaction style and approach of rheumatologists at the Medical University of South Carolina (MUSC) [42,43].

Still, patients noted difficulties with or concerns about planning for the future and managing their illness, particularly among younger patients. Providers may be able to leverage higher levels of trust and positive affect and more engaging visits and conversations to introduce job/career training opportunities, since many patients, particularly African Americans, prefer that their physician makes health decisions [44]. For example, one of the most common concerns among lupus patients is having a doctor who is approachable and listens. Many patients have anxiety about doctor visits because they feel the physician may be callous or not believe they are experiencing unusual symptoms due to lupus. Going to the doctor with a friend or family member is encouraged so the patient will have assistance communicating with the physician [45].

A strength of this study is its focus on a geographical region with a distinct historical environmental context that has the potential to influence healthcare perceptions and decision-making. Thus, these findings deepen our understanding of a unique population with shared historical exposure to racial injustice and pronounced health disparities, particularly with regard to lupus disease activity, damage, and progression [35]. However, a limitation is the small sample size. Moreover, there was no control group in this study. However, this study was designed as an ethnographic exploration of a specific geographical region. The goal was to obtain detailed information from residents on their experiences and perspectives, given the backdrop of a unique historical context. As such, results from this study are not meant to be generalized. Since our target population was residents of Charleston, South Carolina and there was not a formal interview guide, interviews were not translated or their reliability and validity re-evaluated in any other languages, which could limit their use in other contexts as well. Additionally, interviewer bias was possible and self-reported data are subject to recall bias. However, the interviewer was trained in qualitative methodology, respondents were carefully selected, and results were carefully interpreted, yielding noteworthy implications for clinical practice.

## 5. Conclusions

Overall, in the context of a geographical area with a history such as that of Charleston, South Carolina and other areas of the Deep South, trustworthy and competent care can overcome perceptions of doubt, uncertainty, and distrust. Trusted and culturally competent providers are associated with increased medical care and treatment adherence [46]. Based on responses, patient–provider relationships were undoubtedly important to interviewed patients, and the approach adopted by rheumatologists at MUSC could serve as a model for similar geographical areas and patient populations seeking to overcome historical barriers to trust and to improve providers’ communication skills and delivery of disease-specific information.
